# Porous Se@SiO_2_ nanospheres alleviate diabetic retinopathy by inhibiting excess lipid peroxidation and inflammation

**DOI:** 10.1186/s10020-024-00785-z

**Published:** 2024-02-06

**Authors:** Tian Niu, Xin Shi, Xijian Liu, Haiyan Wang, Kun Liu, Yupeng Xu

**Affiliations:** 1 Department of Ophthalmology, Shanghai General Hospital, Shanghai Jiao Tong University School of Medicine, Shanghai, 200080 China; 2 National Clinical Research Center for Eye Diseases, Shanghai General Hospital, Shanghai Jiao Tong University School of Medicine, Shanghai, 200080 China; 3 Shanghai Key Laboratory of Ocular Fundus Diseases, Shanghai, 200080 China; 4 Shanghai Engineering Center for Visual Science and Photomedicine, Shanghai, 200080 China; 5 Shanghai Engineering Center for Precise Diagnosis and Treatment of Eye Diseases, Shanghai, 200080 China; 6 School of Chemistry and Chemical Engineering, Shanghai University of Engineering Science, Shanghai, 201620 China

**Keywords:** Diabetic retinopathy, Porous Se@SiO_2_ nanospheres, Lipid peroxidation, Inflammation

## Abstract

**Background:**

Lipid peroxidation is a characteristic metabolic manifestation of diabetic retinopathy (DR) that causes inflammation, eventually leading to severe retinal vascular abnormalities. Selenium (Se) can directly or indirectly scavenge intracellular free radicals. Due to the narrow distinction between Se’s effective and toxic doses, porous Se@SiO2 nanospheres have been developed to control the release of Se. They exert strong antioxidant and anti-inflammatory effects.

**Methods:**

The effect of anti-lipid peroxidation and anti-inflammatory effects of porous Se@SiO_2_ nanospheres on diabetic mice were assessed by detecting the level of Malondialdehyde (MDA), glutathione peroxidase 4 (GPX4), decreased reduced/oxidized glutathione (GSH/GSSG) ratio, tumor necrosis factor (TNF)-α, interferon (IFN)-γ, and interleukin (IL) -1β of the retina. To further examine the protective effect of porous Se@SiO_2_ nanospheres on the retinal vasculopathy of diabetic mice, retinal acellular capillary, the expression of tight junction proteins, and blood–retinal barrier destruction was observed. Finally, we validated the GPX4 as the target of porous Se@SiO_2_ nanospheres via decreased expression of GPX4 and detected the level of MDA, GSH/GSSG, TNF-α, IFN-γ, IL -1β, wound healing assay, and tube formation in high glucose (HG) cultured Human retinal microvascular endothelial cells (HRMECs).

**Results:**

The porous Se@SiO_2_ nanospheres reduced the level of MDA, TNF-α, IFN-γ, and IL -1β, while increasing the level of GPX4 and GSH/GSSG in diabetic mice. Therefore, porous Se@SiO_2_ nanospheres reduced the number of retinal acellular capillaries, depletion of tight junction proteins, and vascular leakage in diabetic mice. Further, we identified GPX4 as the target of porous Se@SiO_2_ nanospheres as GPX4 inhibition reduced the repression effect of anti-lipid peroxidation, anti-inflammatory, and protective effects of endothelial cell dysfunction of porous Se@SiO2 nanospheres in HG-cultured HRMECs.

**Conclusion:**

Porous Se@SiO_2_ nanospheres effectively attenuated retinal vasculopathy in diabetic mice via inhibiting excess lipid peroxidation and inflammation by target GPX4, suggesting their potential as therapeutic agents for DR.

**Supplementary Information:**

The online version contains supplementary material available at 10.1186/s10020-024-00785-z.

## Introduction

Diabetic retinopathy (DR) is a major microvascular complication of diabetes mellitus (DM), affecting 22.27% of patients with DM (Teo et al. 2021). Retinal endothelial dysfunction is a significant contributing factor in the pathogenesis of DR (Antonetti et al. 2021). As DR progresses, low-grade vascular inflammation and progressive hypoxia can lead to the formation of microaneurysms and acellular capillaries, eventually causing neovascularization (Gui et al. 2020). Vascular endothelial cells are one of the successful treatable targets for DR, with anti-vascular endothelial growth factor (VEGF) therapy being a commonly used treatment for diabetic macular edema (DME). Pathological changes in the vasculature have been partially attributed to elevated VEGF levels. Anti-VEGF therapy has been reported to reduce retinal edema and prevent or reverse neovascularization (Antonetti et al. 2021). However, clinical studies have shown that approximately half of the patients receiving anti-VEGF therapy remain unresponsive, indicating the involvement of other factors in the vascular pathology of DR (Wells et al. 2015). Therefore, new therapeutic strategies are urgently required to complement or replace current anti-VEGF therapies.

Dysregulation of lipid metabolism is a common metabolic alteration in the early stages of DR, which, together with increased reactive oxygen species (ROS) production, can lead to lipid peroxidation (Augustine et al. 2020; Busik 2021). It has been reported to be positively correlated with the duration and severity of diabetes (Kang and Yang 2020). Membrane lipid peroxidation damages the cellular lipid bilayer function and integrity. In addition, byproducts of lipid peroxidation affect nucleic acids and proteins by covalently binding to amino acids or crosslinking the DNA (Gaschler and Stockwell 2017). This process results in the breakdown of the blood–brain barrier by disrupting the endothelial function or integrity (Akhter et al. 2019; Chen et al. 2022a). Increased lipid peroxidation can further cause retinal inflammation by increasing the levels of cell adhesion molecules and activating the nuclear factor (NF)-κB (Dam et al. 2003; Zhong et al. 2019). Chronic retinal inflammation eventually disrupts the blood–retina barrier (BRB) function (Wang and Lo 2018).

Selenium (Se), an essential trace element in humans and animals with various biological functions, is a component of glutathione peroxidase, a cytoprotector against lipid peroxidation (Ingold, et al. 2018). Se is effective in treating inflammatory diseases by reducing the levels of inflammatory cytokines, such as interferon (IFN)-γ, interleukin (IL)-1β, and IL-6 (Rehman et al. 2021). Furthermore, Se intake has been proven to suppress diabetes-induced cell apoptosis and oxidative retinopathy (Daldal and Nazıroğlu 2022). Se supplementation also decreases glucose-mediated oxidative stress in the retinal pigment epithelium (González de Vega et al. 2018). Consistent with previous studies, a cross-sectional study of patients with type 2 diabetes suggested the potential benefits of a Se-rich diet on DR (She et al. 2021).

The application of Se-containing medicine is restricted because of the narrow distinction between its effective and toxic doses. Nanoparticle drug delivery systems offer enhanced solubility and prolonged residence time with fewer side effects for the treatment of retinal diseases (Li et al. 2021). Se nanoparticles (SeNPs) are less toxic and more biocompatible than organic or inorganic Se compounds, attracting the interest of the scientific community (Sun et al. 2014). Porous Se@SiO_2_ nanospheres are innovative nanocomposites that can release Se at a controlled rate to reduce their toxicity and enhance their biocompatibility (Liu et al. 2016). We previously showed that porous Se@SiO_2_ nanospheres can effectively moderate oxidative damage or inflammation in radiation, inflammatory osteolysis, acute lung injury, and prostatic urethral injury (Zhu et al. 2022; Ding et al. 2021; Wang et al. 2020; Yang et al. 2019). However, the potential application of this new nanomaterial in retinal diseases has not yet been investigated. As lipid peroxidation products are a major consequence of oxidative stress, we hypothesized that porous Se@SiO_2_ nanospheres may have therapeutic significance for DR owing to their anti-inflammatory and anti-lipid peroxidation effects. Therefore, in this study, we verified this hypothesis by determining the changes in retinal lipid peroxidation and inflammation in a diabetic mouse model after the intravitreal injection of porous Se@SiO_2_ nanospheres. In addition to the in vivo experiments, we identified the effect of porous Se@SiO_2_ nanocomposites on the inhibition of lipid peroxidation and inflammation in vitro.

## Materials and methods

### ***Synthesis and characterization of porous Se@SiO***_***2***_*** nanospheres***

Porous Se@SiO_2_ nanospheres were synthesized as previously described (Liu et al. 2016). D/max-2550 PC X-ray diffractometer (Cu–Kα radiation; Rigaku; Tokyo, Japan) was used to characterize the phase structure of porous Se@SiO_2_ nanospheres. The average diameter and morphology of the porous Se@SiO_2_ nanospheres were determined using transmission electron microscopy (TEM; JEM-2100F; JEOL, Tokyo, Japan). Porous Se@SiO_2_ nanospheres were suspended in a phosphate-buffered solution (PBS) to obtain a stock solution of 2 mg/mL and stored at 4 °C. Porous SiO_2_ nanospheres (NPs) without Se were used as the controls.

### Cell culture and siRNA transfection

Human retinal microvascular endothelial cells (HRMECs; Cell Systems, WA, USA) were cultured in an endothelial cell culture medium (ScienCell, CA, USA) containing 30 mM D-glucose, 10% fetal bovine serum (FBS; ScienCell), and antibiotics (100 U/mL penicillin and 100 mg/mL streptomycin). The cells were cultured in a 5% CO_2_ incubator at 37 °C.

On reaching 60–70% confluency, HRMECs were transfected with GPX4 siRNA (Silencer select siRNA, ID# s6112; Thermo Scientific, MA, USA) or the negative control, Silencer select negative control #1, in combination with Hyperfect (Qiagen, CA, USA). After 24 h, the medium was changed and Knockdown efficiency was confirmed by Western blotting. Then cells were treated with porous Se@SiO_2_ nanospheres or NPs after maintaining the cells for an additional 24 h. After three days of incubation with a high-glucose (HG; 30 mM d-glucose) medium, further experiments were conducted as described below.

### Cell viability assay

The influence of porous Se@SiO_2_ nanospheres on the viability of HRMECs was evaluated using a cell counting kit (CCK)-8 assay (Dojindo, Kumamoto, Japan). HRMECs were seeded into a 96-well plate at a concentration of 5 × 10^3^ cells/well and treated with porous Se@SiO_2_ nanospheres (0, 20, 40, 60, 80, 100, 120, and 140 μg/mL) for 72 h in a HG medium. Cell viability was measured according to the manufacturer’s instructions.

### Animal study

All animal experiments were approved by the Laboratory Animal Ethics Committee of the Shanghai General Hospital and adhered to the Association for Research in Vision and Ophthalmology guidelines in the Statement for the Use of Animals in Ophthalmic and Visual Research. Male diabetic db/db and control db/m mice were purchased from Jackson Laboratory (*BKS.Cg-Dock7*^*m*+*/*+^ *Lepr*^*db/J*^; ME, USA) and housed in pathogen-free condition under a 12/12 h light/dark cycle with ad libitum access to water and food.

Experiment I: Male mice were randomly assigned to two groups: db/m mice and db/db mice. Retinal samples were collected at 6 months of age. Six mice were used for each group.

Experiment II: Male mice were randomly assigned to four groups: db/m mice, db/db mice, db/db mice treated with porous Se@SiO_2_ nanospheres (db/db + Se@SiO_2_ group), and db/db mice treated with NPs (db/db + NPs group). Thirty mice were used for each group. After the mice were fasted for 6 h, body weight levels were monitored monthly for 2 to 6 months. Corresponding nanospheres (0.5 μg/μL) were intravitreally injected only once into the mice at 1 μL/eye at 3 months of age. db/m and db/db mice were injected with equal amounts of PBS. Retinal samples were collected at 6 months of age.

### Measurement of malondialdehyde (MDA), glutathione, and cytokine levels

Retinal and HRMEC samples were harvested, washed, and lysed according to the manufacturer’s instructions. Protein concentrations were determined using a bicinchoninic acid kit (Sigma-Aldrich, MO, USA). MDA and glutathione concentrations were determined using a lipid peroxidation MDA assay kit, reduced glutathione (GSH) / oxidized glutathione (GSSG) Ratio Detection Assay kit (ab118970, ab138881; Abcam, MA, USA), and a microplate reader. The relative concentrations of MDA and glutathione were calculated by normalizing the measured concentrations to that of the total protein.

### Measurement of cytokine levels

Retinal samples were harvested, washed, lysed, and then determined the protein concentrations. Supernatants were collected from HRMECs. Concentrations of the cytokines, including tumor necrosis factor-alpha (TNF-α), IFN-γ, and IL-1β, were quantified using the enzyme-linked immunosorbent assay (ELISA) kits (MTA00B, MIF00, MLB00C; R&D Systems, MN, USA). To calculate the relative concentrations of cytokines, the measured concentrations were normalized to the total retinal protein.

### Quantitative real-time PCR (qRT-PCR)

Retinal and HRMEC samples were collected and washed. The extraction of total RNA was carried out utilizing Trizol reagent (Invitrogen, Carlsbad, CA, USA), followed by cDNA synthesis using RT Master Mix (Takara, Dalian, China). qRT-PCR analysis was conducted employing an ABI Prism 7500 Sequence Detection System (Applied Biosystems, Foster City, CA), with β-actin serving as the reference gene.

The primer sequences used were as follows: mouse TNF-α forward: CCCTCACACTCAGATCATCTTCT, reverse: GCTACGACGTGGGCTACAG; mouse IFN-γ forward: ATGAACGCTACACACTGCATC, reverse: CCATCCTTTTGCCAGTTCCTC; mouse IL-1β forward: GCAACTGTTCCTGAACTCAACT, reverse: ATCTTTTGGGGTCCGTCAACT; mouse β-actin forward: GGCTGTATTCCCCTCCATCG, reverse: CCAGTTGGTAACAATGCCATGT; human TNF-α forward: CCTCTCTCTAATCAGCCCTCTG, reverse: GAGGACCTGGGAGTAGATGAG; human IFN-γ forward: TCGGTAACTGACTTGAATGTCCA, reverse: TCGCTTCCCTGTTTTAGCTGC; human IL-1β forward: ATGATGGCTTATTACAGTGGCAA, reverse: GTCGGAGATTCGTAGCTGGA; and human β-actin forward: CATGTACGTTGCTATCCAGGC, reverse: CTCCTTAATGTCACGCACGAT.

### Western blotting analysis

Proteins were lysed using the radioimmunoprecipitation assay buffer, separated on 8 or 10% gels via sodium dodecyl sulfate–polyacrylamide gel electrophoresis, transferred to polyvinylidene difluoride membranes, and blocked with 5% bovine serum albumin for 1 h. Then, membranes were incubated with primary antibodies against GPX4 (ab125066; Abcam), ZO-1, occludin, claudin-5 (61–7300, 33–1500, and 35–2500, respectively; Thermo Scientific), and β-actin (3700; Cell Signaling Technology, MA, USA) overnight at 4 °C. After washing, membranes were incubated with horseradish peroxidase-conjugated secondary antibodies (Cell Signaling Technology). ECL western blotting substrate (Millipore, MA, USA) was used to visualize the bands.

### Trypsin digest for retinal vascular architecture

Eyeballs were fixed in 4% paraformaldehyde. Retinas were dissociated and digested in 3% trypsin solution at 37 °C for 30–60 min. Digested retinas were mounted and stained using a Periodic Acid-Schiff Stain Kit (Abcam). Acellular capillaries of the retina were photographed and cells in three high-magnification fields per retinal quadrant were counted.

### Vascular permeability

Mice were anesthetized, intravenously injected with Evans blue (45 mg/kg body weight), and allowed to move for 2 h. Retinas were isolated, dried, weighed, and incubated with Evans blue for 18 h at 70 °C to extract the dye. Absorbance was read spectrophotometrically at 620 nm, and the background absorbance was read at 740 nm. Vascular permeability was calculated as Evans blue per mg of total protein. Evans blue concentration was calculated using a standard curve of the dye in formamide and standardized by the dried retina weight.

### Cell migration assay

After culturing to 90% confluency, HRMECs were wounded to create vertical scratches using a sterile 200-μL pipette tip. The floating cells were removed by washing with PBS and transferred to an FBS-free HG medium. Closure of the denuded zone was photographed using an inverted microscope at 0 and 12 h. The cell migration rate was determined using the NIH ImageJ software.

### Tube formation assay

Approximately 2 × 10^4^ HRMECs were cultured in a Matrigel-precoated 96-well plate (ibidi, WI, USA). After incubation for 6 h, the cells were photographed using an inverted microscope. The tube length and branch points were analyzed using the Angiogenesis Analyzer plug-in in the NIH ImageJ software.

### Statistical analyses

Data were analyzed using Prism 8.4.0 (GraphPad Software, CA, USA) and presented as the mean ± standard deviation. Data from the two groups were analyzed using the Student’s t-test. The normality of multiple groups was analyzed using the Kolmogorov–Smirnov test, whereas the statistical differences were examined using one-way analysis of variance with Bonferroni correction. Body weight change was compared using two-way ANOVA. Statistical significance was set at p < 0.05.

## Results

### ***Structure, characterization, and toxicity of porous Se@SiO***_***2***_*** nanospheres***

**Fig. 1:**
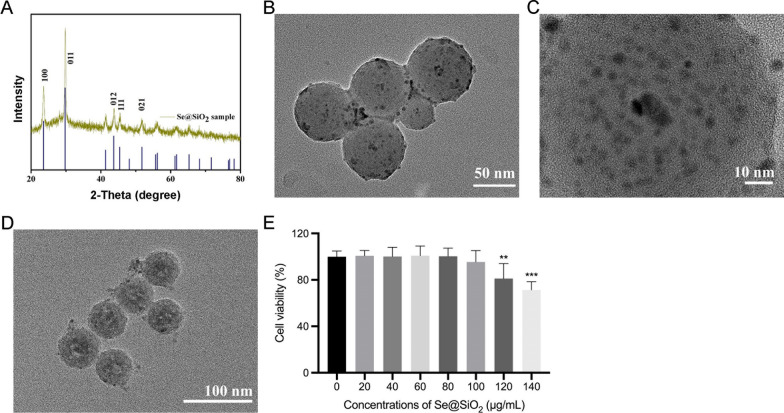
Characterization and cytotoxicity of porous Se@SiO_2_ nanospheres. **A** XRD pattern of porous Se@SiO_2_ nanospheres and standard hexagonal phase of Se (JCPDS card no: 65–1876). **B** Medium-magnification TEM images of the porous Se@SiO_2_ nanospheres. High—**C** and low—**D** magnification TEM images of the porous Se@SiO_2_ nanospheres. **E** Cell viability of HRMECs treated with various concentrations of porous Se@SiO_2_ nanospheres under HG condition (n = 6). Data are represented as the mean ± standard deviation (SD). **p < 0.01, ***p < 0.001

Porous Se@SiO_2_ nanospheres were prepared following a previously reported method (Liu et al. 2016), and their phase structures were determined using X-ray diffraction (XRD). Several characteristic peaks exhibited strong Se signals for the standard Se phase (JCPDS card no 65–1876), including (100), (011), (012), (111), and (021) (Fig. 1A). The XRD pattern of the Se@SiO_2_ nanospheres shows a significant increase in the low-angle region owing to their irregular silica coating. Porous Se@SiO_2_ nanospheres with an average diameter of approximately 55 nm were observed using TEM (Fig. 1B). Porous Se@SiO_2_ nanospheres contained many Se nanocrystals with diameters < 5 nm (Fig. 1C). As shown in Fig. 1D, the Se@SiO_2_ nanospheres became porous after etching with hot water. We used the CCK-8 assay to determine the cellular toxicity of porous Se@SiO_2_ nanospheres on HRMECs under HG conditions. Porous Se@SiO_2_ nanospheres were co-cultured with cells at different concentrations (0–140 μg/mL). Cell viability was unaffected by porous Se@SiO_2_ nanospheres under 100 μg/mL compared to that in the control group (Fig. 1E).

### ***Porous Se@SiO***_***2***_*** nanospheres inhibit excess lipid peroxidation and decrease inflammation***

**Fig. 2:**
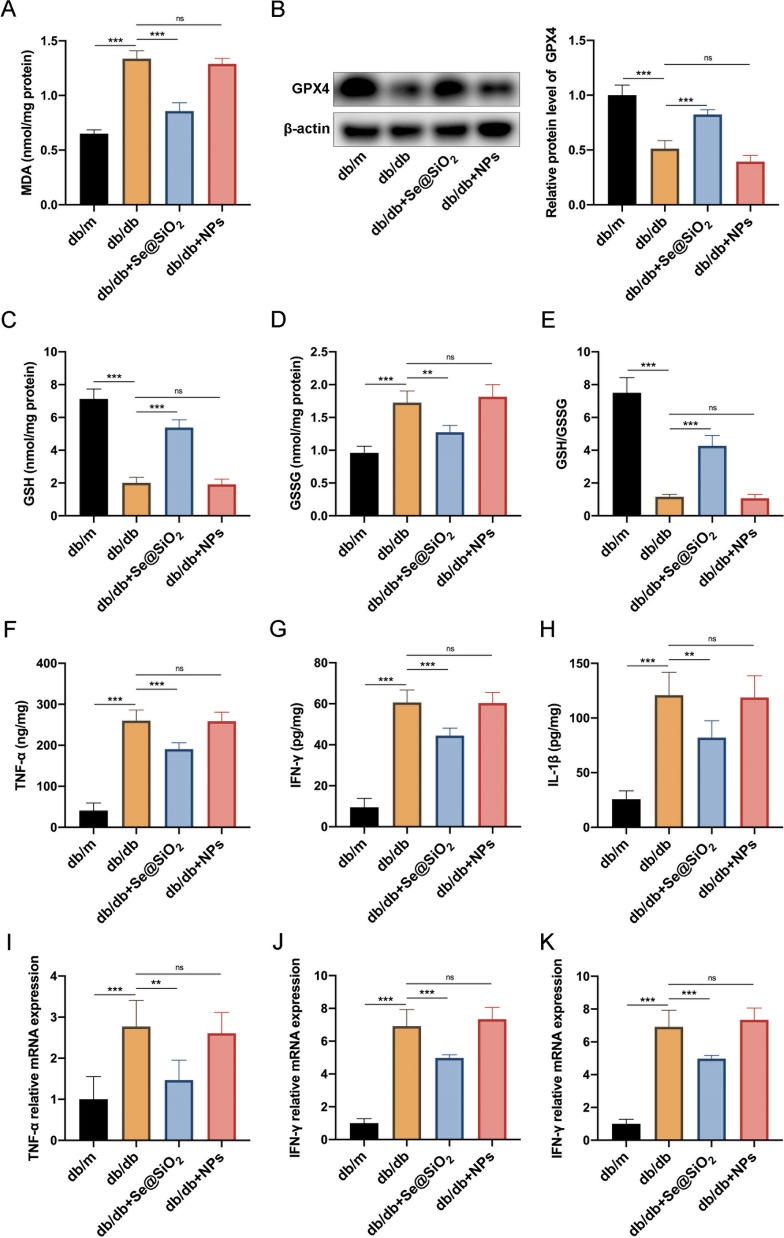
Porous Se@SiO_2_ nanospheres inhibit diabetes-induced retinal lipid peroxidation and inflammation. **A** Levels of MDA in retinal homogenates (n = 6). **B** Expression levels of GPX4 in retinas were measured using western blotting; β‐actin was used as a loading control (left panel). Band densities were assessed using the ImageJ software, and GPX4 expression levels are represented as their ratios to β‐actin (right panel) (n = 3). Levels of GSH (**C**), GSSG (**D**), and the ratio of GSH to GSSG (**E**) in retinal homogenates (n = 6). Protein expression levels of TNF-α (**F**), IFN-γ (**G**), and IL-1β (**H**) in retinal homogenates (n = 6). Relative mRNA expression levels of TNF-α (**I**), IFN-γ (**J**), and IL-1β (**K**) in retinal homogenates (n = 6). Data are represented as the mean ± SD. **p < 0.01, ***p < 0.001; ns, nonsignificant

Extensive lipid peroxidation in diabetes patients accelerates retinal vasculopathy by destroying cellular function and integrity. To assess the therapeutic efficacy of porous Se@SiO_2_ nanospheres in vivo, db/db mice received a single intravitreal injection of porous Se@SiO_2_ nanospheres at 3 months of age, concurrent with the observing the onset of retinal vasculopathy. Furthermore, through the assessment of MDA, GSH, and GSSG levels, and GSH/GSSG ratio, we observed a significant elevation in lipid peroxidation within the retinal tissue of 3-month-old diabetic mice compared to that of the control mice (Additional file 1: Figure S1). Porous Se@SiO_2_ nanospheres have been proposed to address this issue by impeding excessive retinal lipid peroxidation. Their fasting body weight levels were measured monthly from 2 to 6 months of age (Additional file 2: Figure S2). After administering the respective treatments at 3 months of age, samples were collected at 6 months of age. MDA levels increased remarkably in the retinas of db/db mice compared to those of db/m mice and were significantly reduced in the retinas of db/db mice after intravitreal injection of porous Se@SiO_2_ nanospheres (Fig. 2A). Similarly, GPX4 is a selenoenzyme responsible for reducing phospholipid hydroperoxides in membranes to maintain cellular lipid oxidation equilibrium. Western blotting analysis confirmed that db/db mice treated with porous Se@SiO_2_ nanospheres exhibited increased GPX4 protein expression in the retina, which was impaired by DM (Fig. 2B). Moreover, GPX4 oxidizes GSH to GSSG, reducing toxic lipid hydroperoxides (L-OOH) to non-toxic lipid alcohols (L-OH) (Stockwell et al. 2017). Our study also assessed GSH and GSSG levels and GSH/GSSG ratio. Both GSH levels and the GSH/GSSG ratio were significantly lower and GSSG levels were higher in the retinas of db/db mice than in those of db/m mice, and intravitreal injection of porous Se@SiO_2_ nanospheres increased the GSH levels and GSH/GSSG ratio and decreased GSSG levels (Fig. 2C–E). Considering that the byproducts of lipid peroxidation can act as signaling molecules to induce inflammation, levels of multiple inflammatory cytokines were measured in the retinal tissue (Yadav and Ramana 2013). Our study showed that diabetes led to increased TNF-α, IFN-γ, and IL-1β levels, whereas intravitreal injection of porous Se@SiO_2_ nanospheres decreased the levels of these cytokines in db/db mice (Fig. 2F–H). Similar changes in mRNA level were observed by qRT-PCR (Fig. 2I–K). However, there were no significant differences in the indicators between db/db mice injected with PBS and NPs (Fig. 2).

### ***Porous Se@SiO***_***2***_*** nanospheres alleviate retinal vasculopathy in db/db mice***

**Fig. 3:**
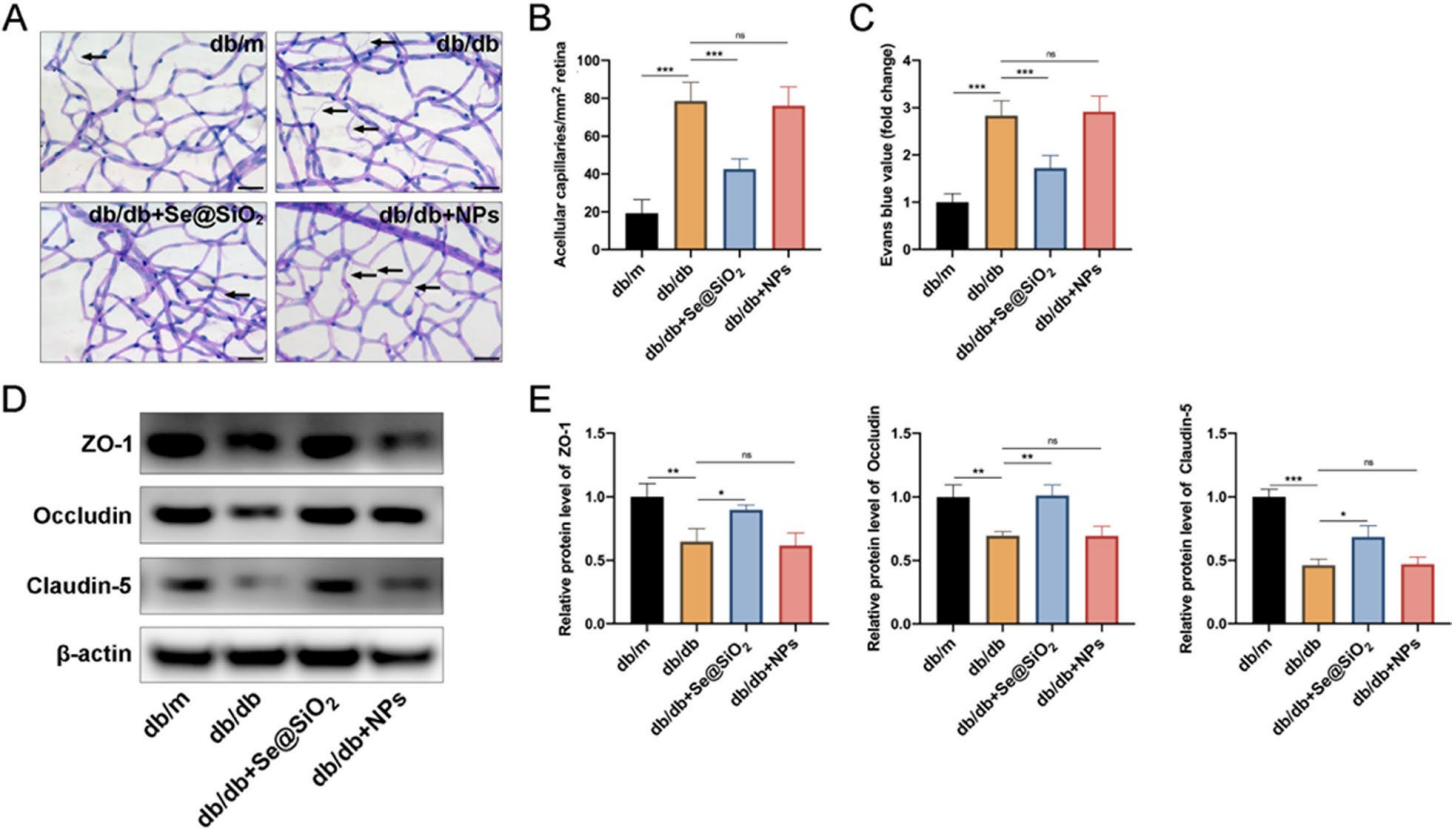
Porous Se@SiO_2_ nanospheres inhibit diabetes-induced retinal vasculopathy. **A** Representative images of the acellular capillaries (arrows) in trypsin-digested retinas. Scale bar, 40 μm. **B** Quantification of acellular capillaries per mm^2^ of retinal area (n = 8). **C** Quantification of Evans blue dye exudation from retinal vessels (n = 6). **D** Expression levels of ZO-1, occludin, and claudin-5 in retinas were measured using western blotting; β‐actin was used as a loading control. **E** Band densities were assessed using the ImageJ software, and the ZO-1 (left panel), occludin (middle panel), and claudin-5 (right panel) expression levels are represented as their ratios to β‐actin (n = 3). Data are represented as the mean ± SD. *p < 0.05, **p < 0.01, ***p < 0.001; ns, not significant

The principal vascular morphological feature of DR is capillary dropout (Feit-Leichman et al. 2005). As expected, the retinas of db/db mice demonstrated more robust accelerated numbers of acellular capillaries than db/m mice, whereas intravitreally injected porous Se@SiO_2_ nanospheres effectively decreased the retinal acellular capillaries in db/db mice (Fig. 3A and B). Evans blue leakage was examined to determine BRB permeability. As shown in Fig. 3C, increased vascular permeability was detected in db/db mice. The db/db mice exhibited a significant decrease in retinal vascular permeability after treatment with the porous Se@SiO_2_ nanospheres. Furthermore, we measured the expression levels of tight junction proteins, ZO-1, occludin, and claudin-5, which play vital roles in maintaining BRB integrity (Niu et al. 2019). Compared with db/m mice, all three tight junction proteins were reduced in the retinas of db/db mice (Fig. 3D and E). Notably, this decrease was reversed by the subsequent porous Se@SiO_2_ nanosphere treatment (Fig. 3D, E). In addition, no difference in retinal vasculopathy was observed between db/db mice intravitreally injected with NPs and PBS (Fig. 3).

### ***Porous Se@SiO***_***2***_*** nanospheres decrease endothelial cell lipid peroxidation and inflammation ***via*** GPX4 ***in vitro

**Fig. 4:**
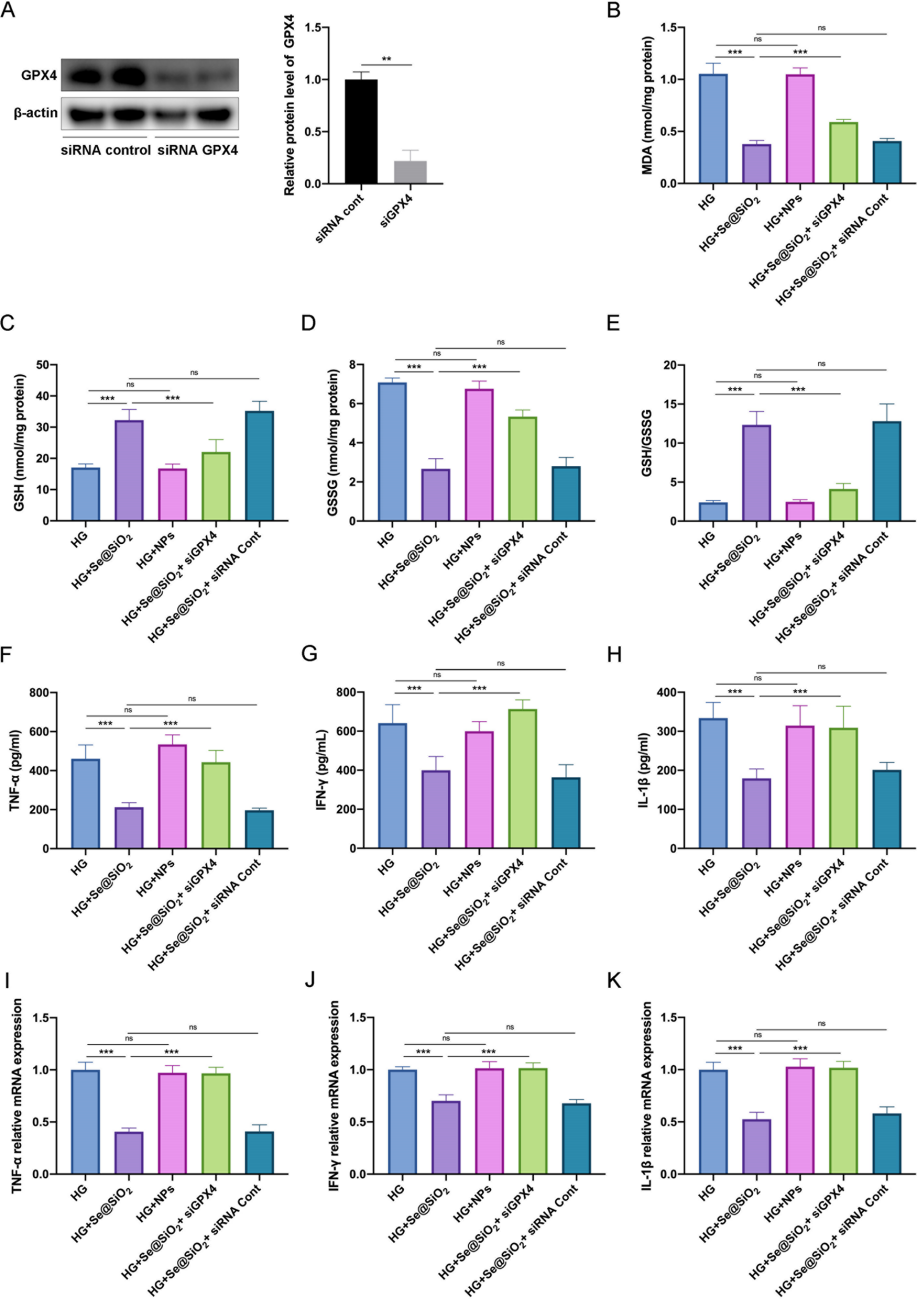
Porous Se@SiO_2_ nanospheres decrease HG-induced lipid peroxidation and inflammation in HRMECs via GPX4. **A** Expression levels of GPX4 in HRMECs transfected with control or GPX4 siRNA was measured using western blotting; β‐actin was used as a loading control (left panel). Band densities were assessed using the ImageJ software, and GPX4 expression levels are represented as their ratios to β‐actin (right panel) (n = 3). Levels of MDA (**B**), GSH (**C**), and GSSG (**D**), and the ratio of GSH to GSSG (**E**) in HRMEC homogenates (n = 6). Protein expression levels of TNF-α (**F**), IFN-γ (**G**), and IL-1β (**H**) in HRMEC homogenates (n = 6). Relative mRNA expression levels of TNF-α (**I**), IFN-γ (**J**), and IL-1β (**K**) in retinal homogenates (n = 6). Data are represented as the mean ± SD. **p < 0.01, ***p < 0.001; ns, not significant

Se exerts antioxidant and pro-survival effects via GPX4 (Friedmann Angeli and Conrad 2018). To test whether GPX4 activates downstream signaling, HRMECs were transfected with siRNA against GPX4 in an HG medium. Transfection of HRMECs with siRNA GPX4 effectively suppressed GPX4 expression (Fig. 4A). Porous Se@SiO_2_ nanospheres suppressed HG-induced lipid peroxidation as indicated by MDA levels and GSH/GSSG ratio, the lipid peroxidation sensor. Porous Se@SiO_2_ nanospheres decreased MDA and GSSG levels while increasing GSH levels and GSH/GSSG ratio (Fig. 4B–E). Conversely, porous Se@SiO_2_ nanospheres showed impaired lipid peroxidation when GPX4 was silenced (Fig. 4B–E). Moreover, porous Se@SiO_2_ nanospheres treatment decreased the levels of TNF-α, IFN-γ, and IL-1β in the supernatants of HRMECs cultured in an HG medium (Fig. 4F–H). Likewise, GPX4 knockdown was found to partly block the ability of porous Se@SiO_2_ nanospheres to mitigate against HG-induced inflammation of HREMCs (Fig. 4F–H). Similar changes in HRMECs endogenous mRNA level were observed by qRT-PCR (Fig. 4I–K). Scrambled siRNAs and NPs had no effect (Fig. 4B–K).

### ***Porous Se@SiO***_***2***_*** nanospheres regulate endothelial cell function ***via*** GPX4 ***in vitro

**Fig. 5:**
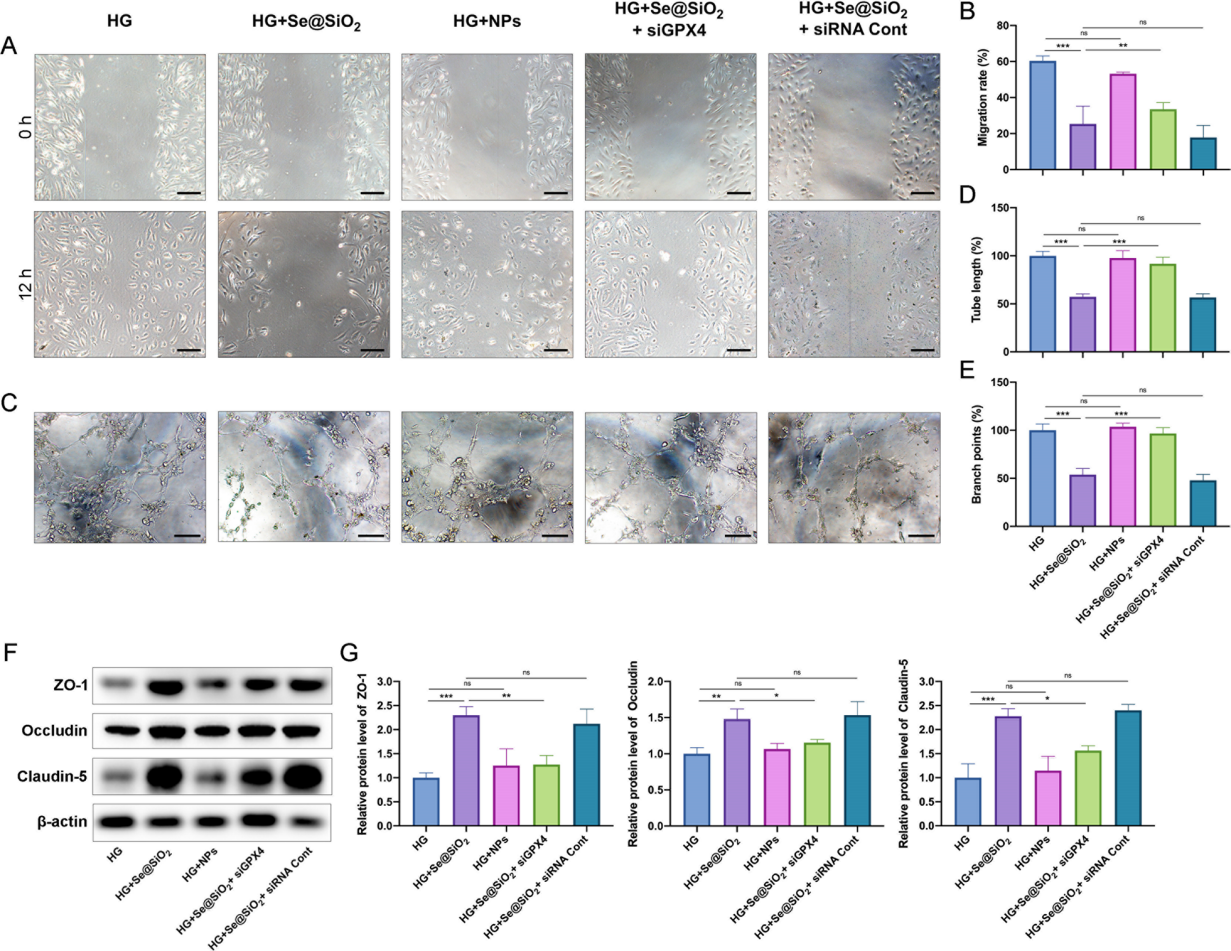
Porous Se@SiO_2_ nanospheres mitigate HG-induced dysfunction of HRMECs via GPX4. **A** Representative images of HRMEC migration at 0 h (top panel) and 12 h (bottom panel). Scale bar, 50 μm. **B** Quantification of HRMEC migration rate (n = 3). **C** Representative images of HRMEC tube formation. Scale bar, 50 μm. Quantification of HRMEC tube lengths (**D**) and branch points (**E**) (n = 6). **F** Expression levels of ZO-1, occludin, and claudin-5 in HRMECs were measured using western blotting; β‐actin was used as a loading control. **G** The band densities were assessed with ImageJ software, and the ZO-1 (left panel), occludin (middle panel), and claudin-5 (right panel) expression levels are represented as their ratios to β‐actin (n = 3). Data are represented as the mean ± SD. *p < 0.05, **p < 0.01, ***p < 0.001; ns, not significant

To determine whether porous Se@SiO_2_ nanospheres regulate HRMECs function by targeting GPX4, siRNA GPX4 was used to decrease GPX4 expression in HRMECs. We first examined the anti-angiogenic effects of the porous Se@SiO_2_ nanospheres on HG-cultured HRMECs. Cells treated with porous Se@SiO_2_ nanospheres showed significantly reduced migration, tube length, and branch points (Fig. 5A–E). Additionally, the porous Se@SiO_2_ nanospheres protected the tight junction-related proteins ZO-1, occludin, and claudin-5 from HG exposure (Fig. 5F and G). GPX4 knockdown decreased the inhibitory effects of porous Se@SiO_2_ nanospheres on HERMC migration and tube formation and protection of tight proteins (Fig. 5). Scrambled siRNAs and NPs had no effect.

## Discussion

Various treatment approaches, such as photocoagulation, intravitreal corticosteroids, anti-angiogenic drugs, and vitreoretinal surgery, have been developed to combat the increased prevalence and burden of DR. However, each treatment has its disadvantages. Although photocoagulation is an effective treatment for peripheral retina with ischemia or retinal proliferation, it can cause adverse side effects, such as peripheral vision loss, choroidal effusion, and macular edema (Reddy and Husain 2018). Owing to their limited durability, intravitreal corticosteroids or anti-VEGF agents require frequent intravitreal injections, thereby increasing the risk of cataracts, retinal detachment, endophthalmitis, and hemorrhages (Solomon et al. 2019). In addition, corticosteroids can induce ocular hypertension or secondary glaucoma (Boyer et al. 2014). Clinical trials revealed that several patients with diabetic macular edema have incomplete responses and suboptimal vision outcomes or require additional photocoagulation (Boyer et al. 2014; Gonzalez et al. 2016). Considering the unmet need for effective treatment strategies, we performed this study to aid in the development of new drugs for DR or DME.

Previous studies have indicated that lipid peroxidation is vital for the occurrence and progression of DR (Kang and Yang 2020). Hyperglycemia causes early lipid oxidation in the retinas of diabetic animals (Torres-Cuevas et al. 2021). Moreover, the end products of lipid peroxidation, lipid aldehydes, are considerably more stable than ROS and cause broader oxidative damage. They are broadly and highly reactive with macromolecules and react with membrane phospholipids to exacerbate oxidative stress (Pamplona 2011). MDA originates from lipid peroxidation of polyunsaturated fatty acids and is thought to be one of the most common mutagenic products of lipid peroxidation (Ayala et al. 2014). Clinical studies have suggested that augmented plasma and vitreous MDA levels are associated with DR progression (Mondal et al. 2022). However, the pathogenic molecular mechanisms of MDA in DR have not been extensively studied until recently. Nonetheless, MDA has proven could induce autophagy dysfunction, VEGF secretion and modify photoreceptor outer segments in age-related macular degeneration, which is recognized as another important blinding retinal disease (Ye et al. 2016; Chen et al. 2022b). Previous studies have reported that Se confers resistance to retinal vasculopathy. For example, Se significantly mitigated retinal microvasculature damage in mice fed a high-sucrose diet (Eckhert et al. 1993). Recently, Se has been shown to reduce lipid peroxidation in the last few years by protecting GPX4 from irreversible inactivation (Ingold et al. 2018). In redox homeostasis, hydroperoxyl phospholipids are metabolized by GPX4 into hydroxyl phospholipids, generating GSSG from GSH. Reductases recycle GSSG to maintain reduced GSH levels within the cells (Stockwell et al. 2017).

Considering that lipid peroxidation plays a vital role in the pathogenesis of DR and the powerful ability of Se to resist lipid peroxidation, we propose that Se is an attractive therapeutic target for DR. However, pro-oxidant and potentially toxic effects can occur if the dose of Se is excessive. Therefore, the widespread use of Se as a clinical drug is limited because of the narrow therapeutic window between its efficacy and toxicity (Khurana et al. 2019). Nanoparticles are considered a wise drug carrier because of their controllable release and stability. Because of these properties, studies have shown that the use of Se via nanoparticles significantly reduces toxicity (Ferro et al. 2021). According to mouse experiments, Che-SeNP has comparable efficacy in upregulating plasma GPX activity to SeMet and selenite but exhibits lower toxicity (Zhang et al. 2005, 2008; Shakibaie et al. 2013). Here, we explored the potential therapeutic effects of porous Se@SiO_2_ nanospheres on retinal vasculopathy in type 2 diabetic mouse model and identified possible mechanisms. We tested the therapeutic effects of the porous Se@SiO_2_ nanospheres on capillary degeneration and BRB disruption. The results showed that diabetic mice treated with an intravitreal injection of porous Se@SiO_2_ nanospheres showed a dramatic improvement in diabetes-induced retinal vasculopathy by significantly inhibiting lipid peroxidation.

In addition to its antioxidant properties, Se has anti-inflammatory and immunomodulatory properties. Se supplementation can decrease the expression levels of main inflammatory cytokines TNF-α, IL-1β, and IL-6 in inflammatory diseases, such as rheumatoid arthritis, atherosclerosis, and colitis (Raza et al. 2022). TNF-α can powerfully induce adhesion molecule expression in endothelial cells and promote inflammation by recruiting leukocytes across the endothelium. Se was able to decrease TNF-α induced expression of adhesion molecules on human umbilical vein endothelial cells (Zhang et al. 2002). In addition, NF-κb is the vital inflammatory signaling pathway that is intimately linked to the inflammatory environment of DR (Rübsam et al. 2018). SeNPs increase the expression and maintain the activity of GPXs (Ferro et al. 2021). And then overexpression GPXs could suppress IκB-α phosphorylation and double IκB-α half-life to inhibit the NF-κb signaling pathway (Raza et al. 2022). Accumulating evidence converges towards DR progression, indicating that exacerbation is strongly associated with inflammation (Rübsam et al. 2018). The vitreous, aqueous humor and serum from diabetic patients with DR show increased levels of inflammatory cytokines and chemokines (Kaštelan et al. 2020). In streptozotocin-induced diabetic rats, increased adherence to leukocytes in the retinal vasculature occurred on the third day after diabetes induction, and a clear spatial correlation of increased leukostasis and BRB impairment was observed (Wang and Lo 2018). Thus, we examined whether the porous Se@SiO_2_ nanospheres showed similar anti-inflammatory effects in the retinas of diabetic mice. Here, we found that intravitreal injecting porous Se@SiO_2_ nanospheres could significantly suppress the expression of TNF-α, IFN-γ, and IL-1β in the retina, which was increased by diabetes. These results confirmed that the porous Se@SiO_2_ nanospheres exerted beneficial effects on diabetes-induced retinal vasculopathy by inhibiting lipid peroxidation and inflammation.

To further decipher the mechanism of action of the porous Se@SiO_2_ nanospheres in the retina under diabetic conditions, we explored their potential downstream targets. HRMECs can be used to further test the effect of porous Se@SiO_2_ nanospheres on endothelial cell function and determine their downstream targets under HG conditions. Consistent with our in vivo observations, we found that the porous Se@SiO_2_ nanospheres exhibited significant effects on HRMECs cultured under HG conditions. Specifically, we observed that the porous Se@SiO_2_ nanospheres effectively suppressed the HG-induced decrease in the expression levels of tight junction proteins, which are crucial for maintaining endothelial barrier integrity. Furthermore, the treatment with porous Se@SiO_2_ nanospheres led to alleviating HG-induced increase of endothelial cell migration and tube formation in vitro, suggesting reduced angiogenic potential under diabetic conditions. Additionally, we observed that the porous Se@SiO_2_ nanospheres exerted a protective effect against lipid peroxidation and inflammation in HRMECs exposed to HG. Lipid peroxidation is a hallmark of oxidative stress, and its inhibition by the nanospheres indicates its antioxidant properties. The suppression of inflammation further highlights the potential of the nanospheres in mitigating the detrimental effects of hyperglycemia on retinal endothelial cells.

GPX4 is one of the most important antioxidant enzymes in the selenoprotein family because it is the only known enzyme that can reduce phospholipid hydroperoxides (Brigelius-Flohé and Maiorino 2013). The present study has identified a noteworthy association between diabetes and diminished expression of retinal GPX4. Prior investigations have elucidated that prolonged exposure to chronic stress and metabolic dysregulation correlates with heightened methylation of the GPX4 promoter, consequently instigating GPX4 inhibition (Liu et al. 2023). This phenomenon could potentially contribute to the observed reduction in retinal GPX4 expression in the context of diabetes. Furthermore, augmented expression of Tripartite motif 46 (TRIM46) in human retinal capillary endothelial cells, induced by elevated glucose levels, has been determined to facilitate the ubiquitination of GPX4. Consequently, this ubiquitination process instigates GPX4 degradation through the proteasome pathway (Zhang et al. 2021). These findings collectively suggest a multifaceted mechanism involving GPX4 promoter methylation and TRIM46-mediated ubiquitination, contributing to the regulation of retinal GPX4 expression under diabetic conditions. The catalytic center of GPX4 contains selenocysteine, which contributes to the expression and activity of GPX4 (Ingold, et al. 2018; Sunde 2018). Through siRNA-mediated GPX4 knockdown experiments, we discovered that the protective effects of the porous Se@SiO_2_ nanospheres on lipid peroxidation, inflammation, and endothelial cell function were partially dependent on GPX4. This finding suggests that GPX4 may be one of the downstream targets of the porous Se@SiO_2_ nanospheres, through which they exert their beneficial effects on retinal endothelial cells under diabetic conditions. Based on our findings, it is postulated that the introduction of porous Se@SiO_2_ nanospheres facilitates the gradual and sustained release of Se, a critical element for the activity of GPX4. As a result, the enhanced availability of Se promotes the upregulation of GPX4 activity, amplifying its capacity to detoxify lipid hydroperoxides and maintain cellular redox homeostasis. The effective control of lipid peroxidation by GPx4 serves as a preventive measure against ferroptosis induction. Furthermore, GPX4 can inhibit inflammatory responses by suppressing excessive inflammasome activation, leukotrienes, prostaglandin D2, and other inflammatory mediators, as well as by reducing the expression of adhesion molecules involved in inflammatory events (Ursini et al. 2022). This mechanism plays a crucial role in safeguarding retinal endothelial cells against oxidative damage and inflammation, thereby preserving their overall cellular functionality to suppress diabetic-induced retinal vasculopathy.

## Conclusion

DR is a major cause of visual impairment and blindness among individuals in the working-age population. In a previous study, our research group developed porous Se@SiO_2_ nanospheres as a means of delivering sustained Se release (Liu et al. 2016). Building upon this work, our current findings demonstrate the remarkable therapeutic efficacy of porous Se@SiO_2_ nanospheres in combating DR-induced vasculopathy. Notably, we discovered that this therapeutic effect is partially mediated by the targeting of GPX4. In summary, our study highlights the promising potential of porous Se@SiO_2_ nanospheres as a valuable candidate for clinical treatment of DR. This research paves the way for innovative approaches to targeted and sustained drug delivery, offering hope for improved outcomes and a brighter future for individuals affected by DR-related visual impairment and blindness.

### Supplementary Information

**Additional file 1: Figure S1.** Diabetes-induced retinal lipid peroxidation. (A) Levels of MDA in retinal homogenates (n = 6). Levels of GSH (B), GSSG (C), and the ratio of GSH to GSSG (D) in retinal homogenates (n = 6). Data are represented as the mean ± SD. ***p < 0.001.

**Additional file 2: Figure S2.** Body weight changes of each group (n = 30). Data are represented as the mean ± SD. ***P < 0.001.

## Data Availability

All data in the article is available from the corresponding author upon reasonable request.

## Abbreviations

DR: Diabetic retinopathy
Se: Selenium
GPX4: Glutathione peroxidase 4
DM: Diabetes mellitus
VEGF: Vascular endothelial growth factor
ROS: Reactive oxygen species
NF: Nuclear factor
BRB: Blood-retina barrier
IFN: Interferon
IL: Interleukin
SeNPs: Se nanoparticles
XRD: X-ray diffraction
TME: Transmission electron microscopy
PBS: Phosphate-buffered solution
HRMECs: Human retinal microvascular endothelial cells
FBS: Fetal bovine serum
CCK: Cell counting kit
HG: High-glucose
MDA: Malondialdehyde
GSH: Reduced glutathione
GSSG: Oxidized glutathione
TNF: Tumor necrosis factor
ELISA: Enzyme-linked immunosorbent assay
L-OOH: Lipid hydroperoxides
L-OH: Lipid alcohols
